# Host-Size Mediated Trade-Off in a Parasitoid *Sclerodermus harmandi*

**DOI:** 10.1371/journal.pone.0023260

**Published:** 2011-08-11

**Authors:** Zhudong Liu, Bingbing Xu, Li Li, Jianghua Sun

**Affiliations:** 1 State Key Laboratory of Integrated Management of Pest Insects and Rodents, Institute of Zoology, Chinese Academy of Sciences, Beijing, China; 2 School of Life Sciences, Guizhou Normal University, Guiyang, China; Ghent University, Belgium

## Abstract

Optimality models of host-parasitoid relationships have traditionally assumed that host quality increases as a function of host size at parasitism. However, trade-offs may play a crucial role in species evolution and should be found in host-parasitoid interactions where the host quality may differ between different sizes. Here, we investigated the effects of host size differences of *Monochamus alternatus* larva on foraging decisions, parasitism and related fitness in a gregarious ectoparasitoid, *Sclerodermus harmandi*. Two-choice and non-choice experiments were conducted with *M. alternatus* larvae to evaluate preference and performance of *S. harmandi*, respectively. Results from two-choice test showed that adult females prefer to attack large larvae rather than small larvae. In no-choice tests, adult females needed more time to paralyze large larvae than small larvae before laying eggs on the body surface of *M. alternatus* larvae and had lower survival and parasitism rate on those large larvae. Foraging decisions of *S. harmandi* led to the selection of the most profitable host size for parasitoid development, which showed more offspring gained on large *M. alternatus* larvae than on small larvae and got higher body weight on those large hosts. This study demonstrates the existence of trade-off occurring during host-parasitoids interactions according to host size related quality.

## Introduction

For insect parasitoids, the host represents the sole nutritional and physiological environment during immature development. Host quality evaluation by female parasitoids should play a key role with host choice resulting in trade-offs due to variation in host quality and developmental requirements [1], [2], [3]. Typically, trade-offs play a key role in host selection since the host quality may differ between host age categories [1], [4]. These hosts differ in age, body size, behavioral and physiological and immunological status, and thus represent resources of varying qualities and quantities [5]. Nutritional quantity of a host is presumably determined by the host size and the amount of host tissues available for parasitoid development [5], [6], [7].

In the context of host selection, parasitoids that forage optimally should adopt behaviors that provide the highest fitness return or profitability in relation to the host size or age distribution [1], [4]. Many parasitoids are able to assess the quality of hosts through host size and selectively parasitize hosts of a certain size [5], [8]. Host stage-selective feeding and oviposition reduce competition for hosts between adult females and their progeny or among progeny, with a corresponding increment in offspring survival and performance [9]. For the majority of ichneumonid and many braconid parasitoid wasps, larvae consume most or all of the host tissues prior to pupation [10] and host size translates directly into parasitoid body size [1]. Body size, in turn, is generally expected to have a positive correlation with the fitness of the eclosing parasitoid [11]. One of the optimal patterns emerging from previous studies on the life history strategies of parasitoids is that large body size confers greater fitness [1] and closely correlates with the stage of the host at parasitism [12], [13].

*Sclerodermus harmandi*, a bethylid hymenoptera (Hymenoptera: Bethylidae), is one of most widely used parasitoids in controlling *Monochamus alternatus* (Coleoptera: Cerambycidae), the most important vector of the pinewood nematode, *Bursaphelenchus xylophilus* Steiner et Buhrer in Japan and China [14], [15]. *S. harmandi* is a synovigenic anautogenous species (i.e., oogenesis takes place after females feed on hosts and are stimulated by direct access to suitable hosts for oviposition) [9]. The pre-oviposition period usually lasts several days according to host species. It has been reported that *S. harmandi* parasitoid acquires favorable attributes due to the effect of variable host resources on behavioral preference and subsequent adult/offspring fitness [16], [17], [18]. Furthermore, *S. harmandi* likely requires effective searching tactics in finding its hosts, which tend to be solitary wood-boring insects in cryptic situations (wattle, tree trunks, wood and seeds) [19], [20], [21].

*S. harmandi* is a gregarious idiobiont parasitoid in which females permanently paralyze the attacked hosts before eggs are laid on them. Thus, the amount of resource available is critical important for both the adult and progeny fitness. Although the parasitoids have a wider range of suitable host species, there is a trade-off between higher fitness gain and lower parasitism rate [4]. Parasitoid fitness is usually measured by life-history traits such as development time, survival, fecundity, sex ratio, and size [1], [22], and several host quality models assume that fitness is related to host size or age at parasitism [6], [23]. It has been shown that progeny emerging from larger hosts tend to be positively correlated to fitness parameters, such as fecundity and survival of parasitoids [11]. Moreover, host quality can influence sex allocation patterns in arrhenotokous parasitoids [5], [12], in which sons develop from unfertilized haploid eggs by parthenogenesis while daughters develop from fertilized (diploid) eggs by gamogenesis. *S. harmandi* reproduces arrhenotokously and has a wide range of suitable host species. Hosts include mainly from some beetles and hymenopterans with ample size variation [24]. *M. alternatus* is one the most abundant host species [24]. Size of *M. alternatus* larvae ranges from 200 mg to 700 mg in field wild (personal observation), providing parasitoids a wide choice of different size hosts to attack. Parasitoids are expected to attack larger hosts since they contain a greater quantity of resources than small or juvenile hosts; thus trade-off between fitness gain and host size are expected.

Here, we report how well the response of *S. harmandi* to host size matches the suitability of hosts for subsequent parasitoid fitness-related performances. Under laboratory conditions, feeding preference of *S. harmandi* females between two sizes host of *M. alternatus* larvae was compared. Effects of host size on performance of both mother and offspring of *S. harmandi* were investigated. Regression analysis was used to evaluate the relationship between parasitoid fitness and host size, combined parasitoid mother performance, to explore the host size mediated trade-off in host use by parasitoid *S. harmandi*.

## Results

### Parasitoid behavioral response to host size

**Figure 1:**
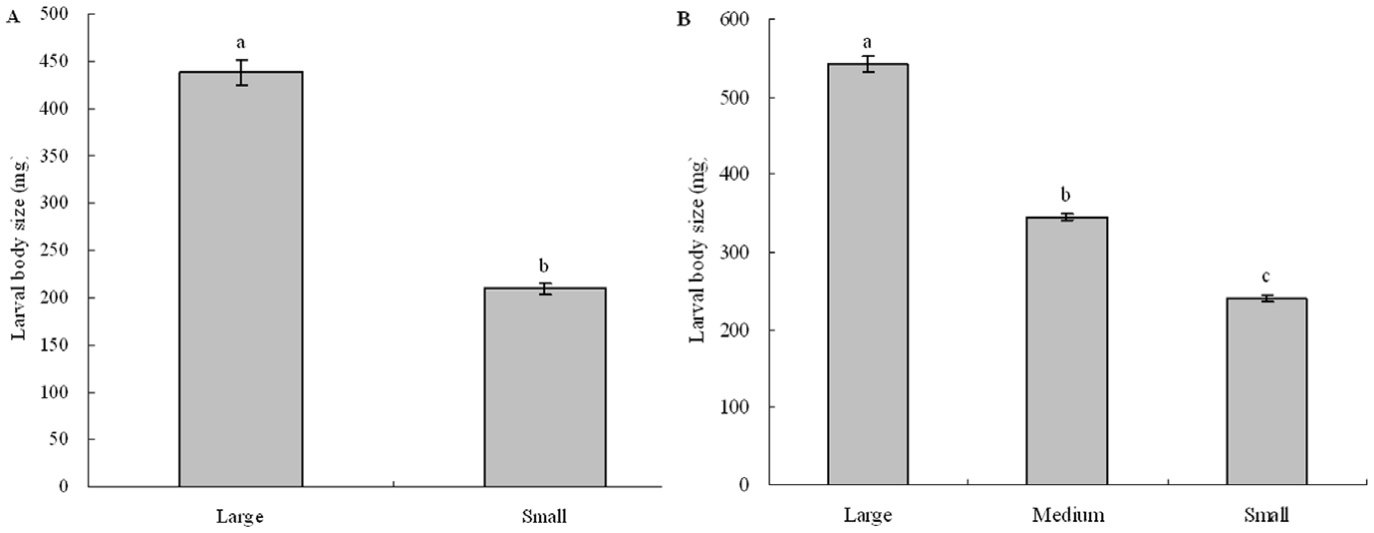
Body size difference of *Monochamus alternatus* larvae for dual-choice and no-choice tests of *Sclerodermus harmandi*. (A) beetle larvae for dual-choice test; (B) beetle larvae for no-choice test. Bars indicate standard errors and different letter on the bar means significantly different at p≤0.05 (ANOVA) (A) and p≤0.05 with Bonferroni Multiple Comparison (ANOVA) (B).

**Figure 2:**
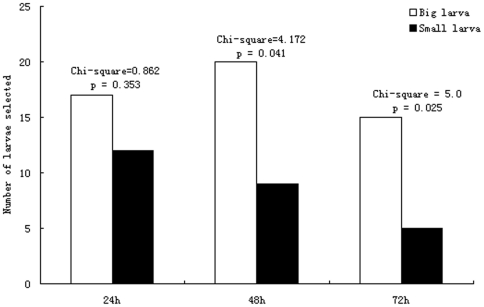
Attacking preference of *Sclerodermus harmandi* between large and small *Monochamus alternatus* larvae in 72 h.

There was a significant difference between large and small groups of host *M. alternatus* larvae with average weight of 438.4±13.1 mg and 209.4±5.9 mg respectively (Figure 1A: ANOVA, F_1, 90_ = 252.354, *p*<0.0001). When these two host sizes of *M. alternatus* larvae were exposed simultaneously of *S. harmandi* females, the wasp significantly preferred the large ones rather than the small ones, starting 48h after exposure (Figure 2: 24h, Chi-square = 0.862, *p* = 0.353; 48h, Chi-square = 4.172, *p* = 0.041; 72h, Chi-square = 5.0, *p* = 0.025).

### Adult performance and host size

In no-choice tests, the three groups of *M. alternatus* larvae had significantly different body mass (Figure 1B: F _2, 144_ = 456.442, *p*<0.0001). Averages for each group were: 541.8±10.33 mg (large), 344.9±3.57 mg (medium) and 240.3±3.39 mg (small), respectively.

**Figure 3:**
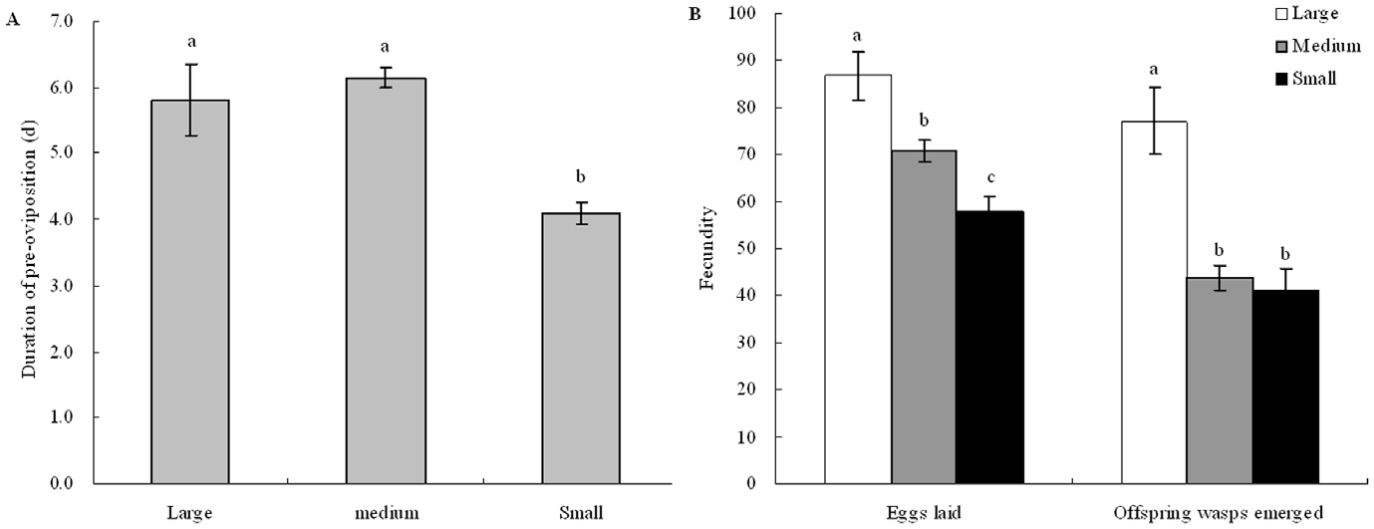
Duration of pre-oviposition and fecundity of *Sclerodermus harmandi* females on *Monochamus alternatus* larvae. (a) Duration of pre-oviposition of adult females; (b) female fecundity. Bars indicate standard errors and different letter on the bar means significantly different at p≤0.05 with Bonferroni Multiple Comparison (ANOVA).

Adult performance of female wasps varied significantly among host size class (Table 1), showing as mortality (Chi-square = 6.203, *p* = 0.045) and parasitism rate (Chi-square = 9.105, *p* = 0.011). Wasps had much higher mortality attacking large larvae compared to small larvae (Chi-square = 5.92, *p* = 0.023), and had lower parasitism rate on large larvae over small larvae (Chi-square = 7.94, *p* = 0.005). However, once larvae were oviposited by female wasps, all sizes of *M. alternatus* larvae could support parasitoid larvae development to adult showing no differences among large, medium and small treatments (Table 1: Chi-square = 2.725, *p* = 0.256). When *M. alternatus* larvae were exposed separately to females, shorter periods of pre-oviposition were observed on small size larvae than on large and medium size larvae (Figure 3A: F_2, 75_ = 14.793, *p*<0.0001). Female fecundity (both number eggs laid and offspring wasp emerged) was significantly higher on the large larvae than small larvae of *M. alternatus* (Figure 3B: eggs laid, F_2, 72_ = 13.207, p<0.0001; offspring wasp emerged, F_2, 55_ = 13.698, *p*<0.0001). Besides the difference in wasp performance, various *M. alternatus* larvae showed different characteristics, which indicated that small *M. alternatus* larvae were prone to die of desiccation compared with large *M. alternatus* larvae (Chi-square = 4.352, *p* = 0.037), although it was slightly significant difference among large, medium and small (Table 1: Chi-square = 5.330, *p* = 0.07).

**Table 1:** Costs of *Sclerodermus harmandi* attacking *Monochamus alternatus* larvae.

	Number of				
	Replicates	Wasps dead	Wasps who laid eggs	Wasps whose offspring emerged	Larvae dried dead
Large larvae	50	18/50 a	16/32 a	12/16 a	3/50 a
Medium larvae	46	9/46 ab	28/37 b	24/28 a	4/46 ab
Small larvae	50	8/50 b	34/42 b	22/34 a	10/50 b
Chi-square		6.203	9.105	2.725	5.330
*p*		0.045	0.011	0.256	0.070

Different letter means significantly different at p≤0.05 (Chi-square test).

**Figure 4:**
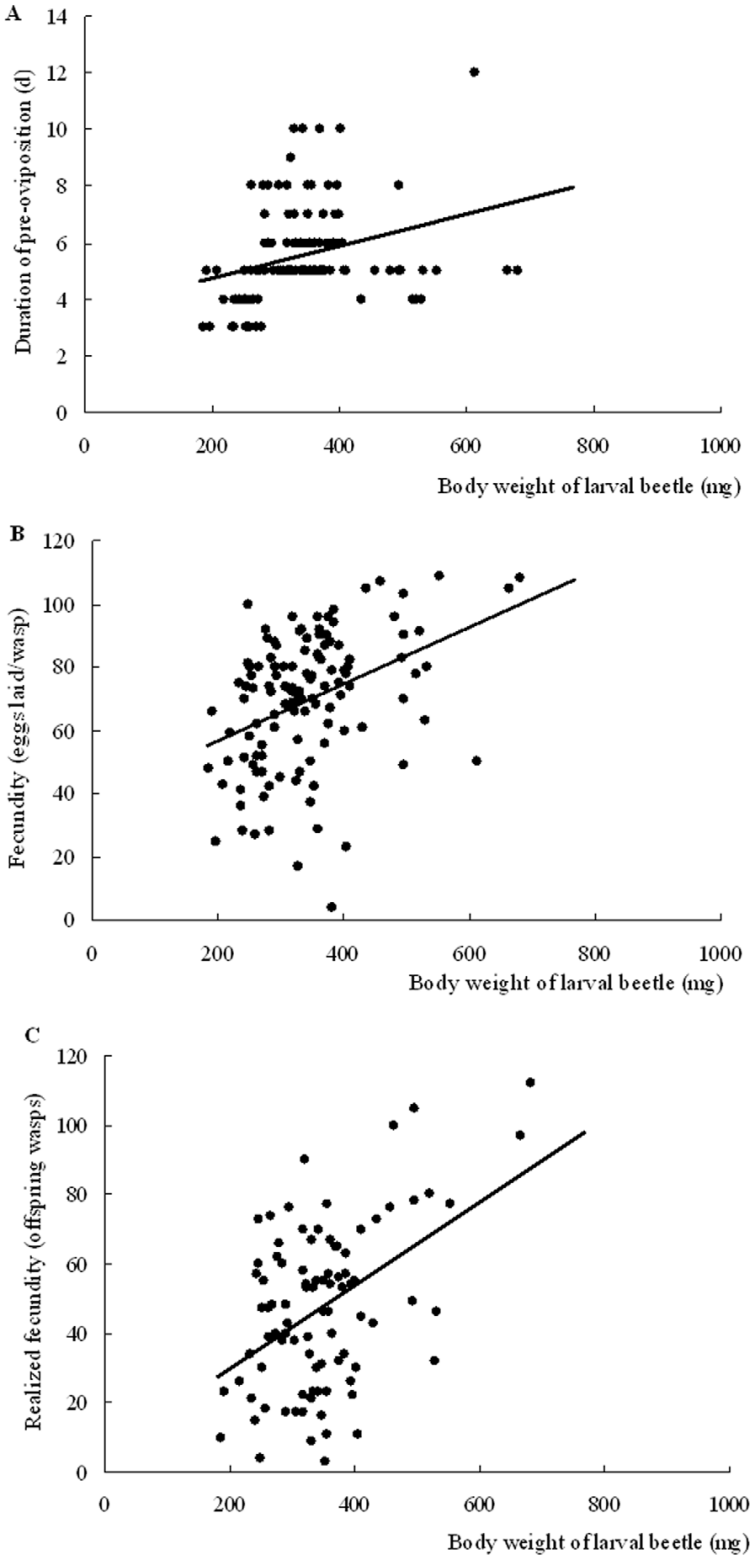
Correlation between *Monochamus alternatus* larvae size and *Sclerodermus harmandi* mother wasp fitness indices. Effect of host size is assessed separately for (A) duration of pre-oviposition (y = 0.0056×+3.6497, r^2^ = 0.0955, F_1,129_ = 13.623, *p*<0.0001), (B) egg laid (y = 0.0891×+39.075, r^2^ = 0.1529, F_1,120_ = 21.664, *p*<0.0001), and (C) offspring wasps emerged (y = 0.1203×+5.6948, r^2^ = 0.2142, F_1,90_ = 24.527, *p*<0.0001).

Host body size was significantly correlated with the duration of pre-oviposition (Figure 4A: *r* ^2^ = 0.0955, F_1, 129_ = 13.623, *p*<0.0001). Furthermore, fecundity was also significantly correlated with host body size, which showed the large hosts supported more offspring wasp (Figure 4B: eggs laid, *r* ^2^ = 0.1529, F_1, 120_ = 21.664, *p*<0.0001; Figure 4C: wasps emerged, *r* ^2^ = 0.2142, F_1, 90_ = 24.527, *p*<0.0001).

### Offspring performance and host size

**Figure 5:**
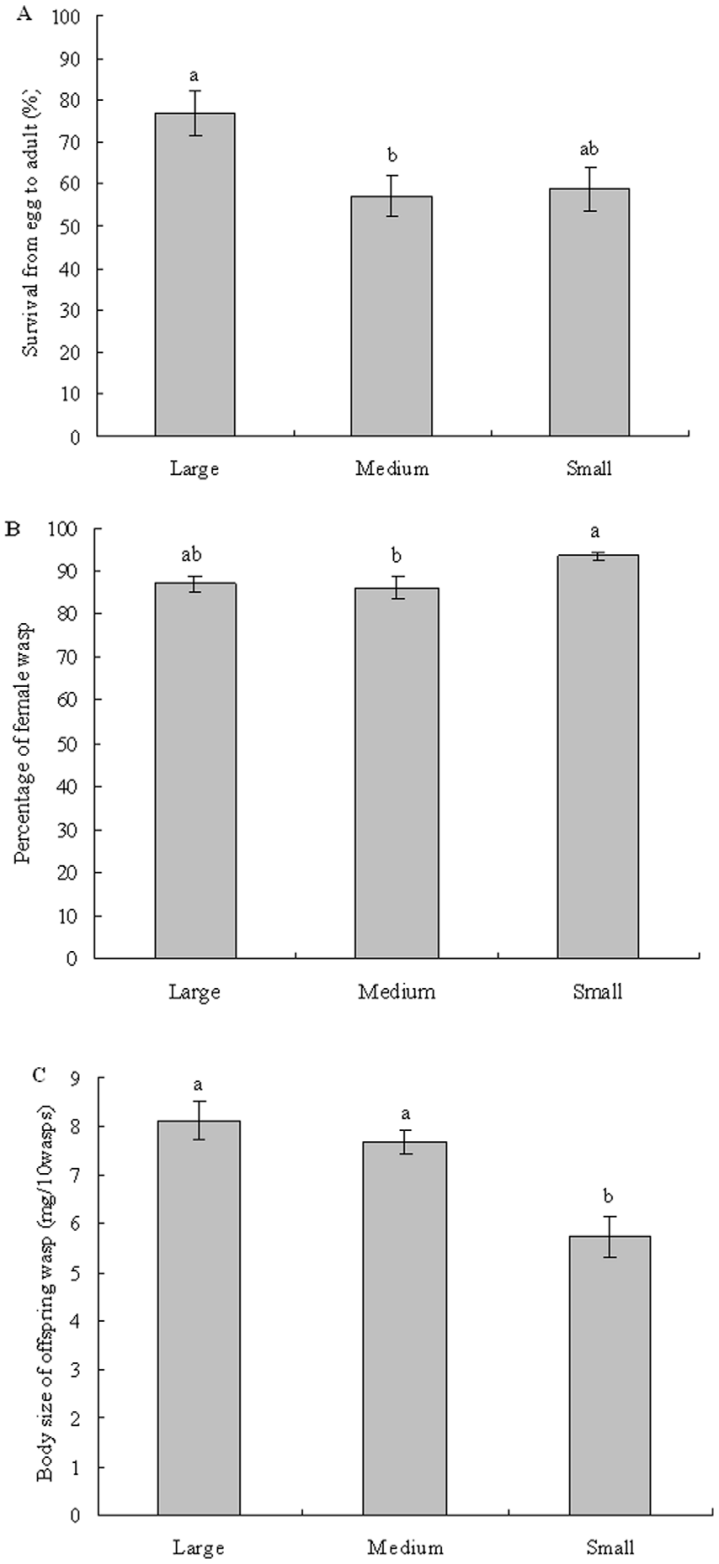
Offspring performance of *Sclerodermus harmandi* from various size of *Monochamus alternatus* larvae. (A) offspring survival, (B) sex ratio (proportion of females), and (C) offspring wasp body size measured as weight. Bars indicate standard errors and different letter on the bar means significantly different at p≤0.05 with Bonferroni Multiple Comparison (ANOVA).

The wasp offspring had higher survival on large larvae of *M. alternatus* (Figure 5A F_2, 53_ = 4.239, *p* = 0.018). Offspring sex ratio of the two colonies was female-biased and differed among large, medium and small *M. alternatus* larvae. Wasp developing on small hosts had higher percentage of offspring female (Figure 5B; *F*_2, 54_ = 4.806, *p* = 0.012). Furthermore, adult offspring developing on large and medium *M. alternatus* larvae were heavier than those developed on small larvae (Figure 5C; ANOVA; F _2, 53_ = 11.356, *p*<0.0001).

**Figure 6:**
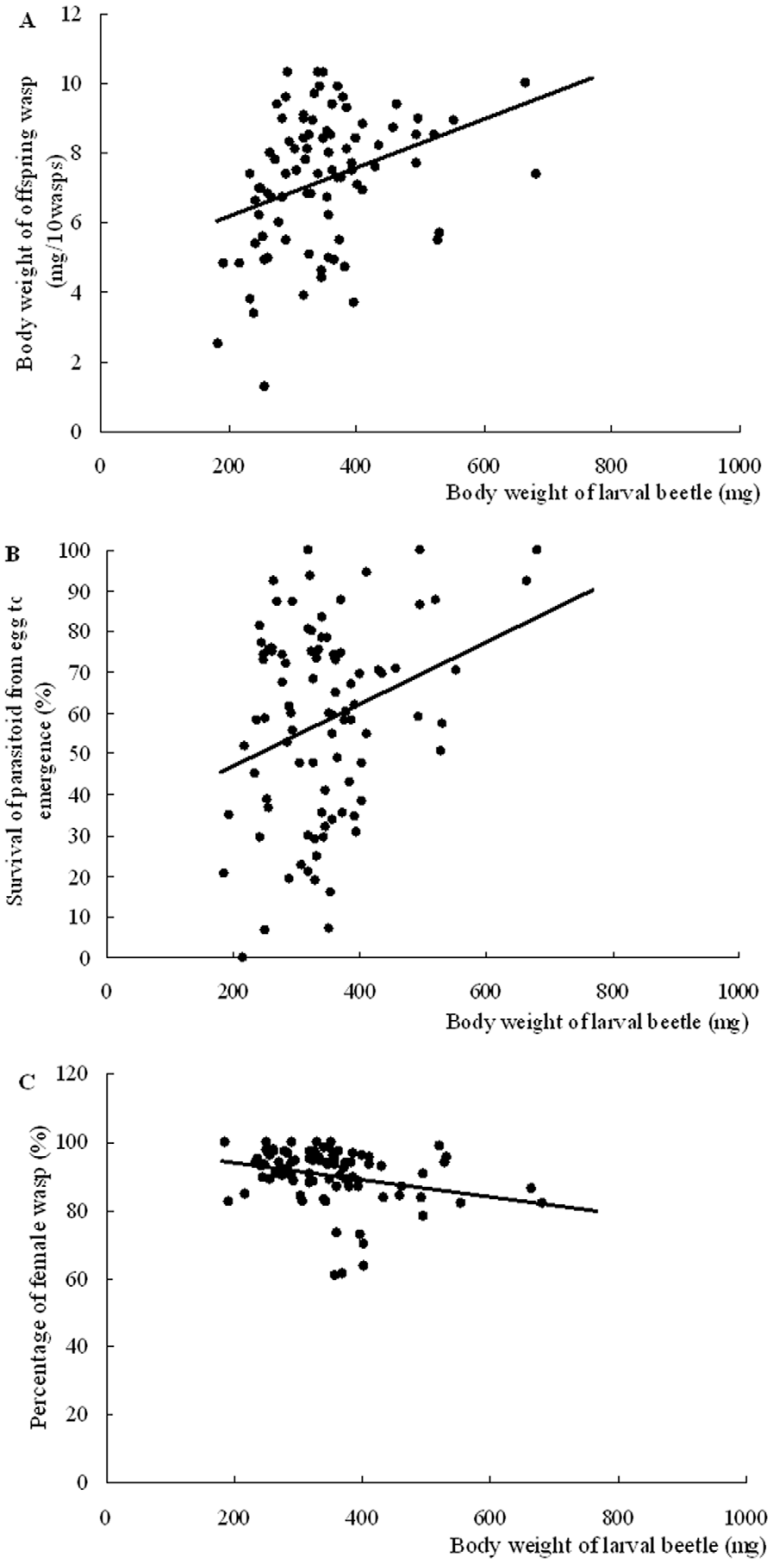
Correlation between *Monochamus alternatus* larvae size and *Sclerodermus harmandi* offspring fitness indices. Effect of host size is assessed separately for (A) offspring body size (y = 0.0069×+4.8103, r^2^ = 0.0.113, F_1,84_ = 10.706 *p* = 0.002), (B) offspring survival (y = 0.0761×+31.913, r^2^ = 0.086, F_1,88_ = 8.299, *p* = 0.005), and (C) Offspring sex ratio (female percentage) (y = −0.0246×+98.916, r^2^ = 0.0764, F_1,85_ = 7.035, *p* = 0.01).

Offspring parasitoid survival and body weight were regressed with the host size, showing that host body size was significantly correlated with offspring fitness indices (Figure 6). Host body size was significantly correlated with offspring body weight of parasitoid, which showed the large host produced large offspring wasp (Figure 6A: *r* ^2^ = 0.113, F_1, 84_ = 10.706, *p* = 0.002). Offspring survival from egg to emergence was significantly correlated with host body size (Figure 6B: *r* ^2^ = 0.086, F_1, 88_ = 8.299, *p* = 0.005). Moreover, sex ratio measured as female percentage was significantly correlated with host size, which shows that female percentage decreases with the increased host size (Figure 6C r^2^ = 0.0764, F_1,85_ = 7.035, *p* = 0.01).

## Discussion

Parasitoid wasps have been used as model organisms for the study of life history evolution [1], [25], [26], [27]. The preference and performance of *S. harmandi* on different sizes of *M. alternatus* larvae were estimated to ascertain whether female host selection and subsequent fitness corresponded with host quality. *S. harmandi* females preferred to attack large larvae of *M. alternatus* but needed to spend more time to deal with the large host before oviposition, incurring a higher risk of adult mortality and lower parasitism rate. Subsequently, their offspring gained higher body weight and survival on those large larvae. The capacity of *S. harmandi* females to grow on various size host larvae suggests an ability to select large host and gain advantage for offspring, which clearly supports the hypothesis of optimal oviposition theory [12], [18], [28]. However, since the large larvae of *M. alternatus* are more powerful and active, they could respond actively by attacking the invaders when bit by wasps; more venom secreted by parasitoids is would be then needed to induce paralysis, which could decrease adult wasp's fitness. This is the first evidence on a host use trade-off mediated by host size.

The effect of maternal host/colony is not replicated as all parasitoid individuals come from the same long-term colonies and were kept in the laboratory for 20 generations on *M. alternatus* larvae. Any differences found between host sizes are not necessarily due to the maternal host, it could be caused by other reasons associated with host size. For most parasitoid wasps, successful parasitism is associated with multiple trade-offs between different behavioral and physiological constraints. Indeed, it was reported that host size determines host's behavioral and physiological defense, particularly where juvenile parasitoids consume virtually all host tissues before pupation [1], [13], [29], [30].

*M. alternatus* larvae exhibited behavioral defenses to parasitoid attacks, shaking their body and biting the attackers. These defense behaviors have been shown to differ between large and small larvae, with small larvae being far less active and lacking strong mandibles to defend themselves than large ones (personal observation). On the other hand, host's immune system, metabolism and nutritional status, changes with development can influence the quality of immature hosts. This results in lower fitness and has been shown in aphids *Toxoptera citricida* when parasitized by *Lipolexis oregmae* [31]. In this study, wasps prefer to select large larvae. Besides large larvae defend more actively, large larvae are not easier to be paralyzed by the same amount of venom secreted by parasitoids when comparing with small larvae. This poses some disadvantages, longer handing time before oviposition, higher adult wasp mortality and lower parasitism rate were observed on large *M. alternatus* larvae.

Why would wasp evolve preference for large *M. alternatus* larvae if large larvae can actively defend parasitoids attacks by shaking their body and are not easy to be paralyzed by the venom secreted by parasitoids? For gregarious idiobiont parasitoids, which often kill or paralyze the attacked hosts, there is a trade-off between higher fitness gain and lower parasitism rate [4]. Parasitoids are expected to attack larger or near mature hosts, which contain a greater quantity of resources than small or juvenile hosts. Progeny that emerge from larger hosts is presumed upon benefiting from increased adult size that tends to be positively correlated to fitness parameters [11]. Since large hosts could bring lots of benefits for the parasitoids, the parasitoids could develop well on large size host and adapt to large hosts under natural selection pressure through long time adaptation. In field, the wasp *S. harmandi* may encounter several beetle species ranging in body size from about 30 mg to 700 mg such as *S. populnea* and *M. alternatus*. Host weight even differs in the same species with different age such as *M. alternatus*, which would allow the wasp to assess the host quality and make a decision. In the current study, it is presumed that large larval hosts contain more nutrients which could support more offspring wasps on them. Indeed adult wasps got more fecundity (both eggs laid and offspring wasp emerged) and higher offspring survival, which all are positive lineal relationship with host size.

Moreover, host quality is known to influence sex allocation in many parasitoid species by producing more females where females are allocated to hosts of a higher quality [5], [32], [33], [34], since the fitness of sons suffers less from being small than the fitness of daughters who will have to produced eggs in turn [35]. However, our results do not support the host quality model suggested by Charnov and Skinner [33], in which percentage of female is negative correlated with host body size. This is also shown in a koinobiont aphid parasitoid that the occurrence of more males in the larger fourth instars and adults [4]. Other characteristics such as physiological immunity and behavioral defense should also be considered to influence sex ratio of *S. harmandi* [35], [36]. In the current study, the occurrence of more males in increased offspring wasps gained on large host could be indicated as a kind of adaptation. As known, *S. harmandi* reproduces arrhenotokously and the female is able to influence the progeny sex ratio (proportion of males in the population) at oviposition by regulating fertilization of the eggs. Gamogenesis brings more fitness than parthenogenesis, which would produce both sexes to lay more female offspring rather than males only. As host size increased, more female wasps produced needs enough males to guarantee to mate completely with all females to produce more female offspring, which could contribute maximally to progeny fitness.

Theoretical and practical work shows that large size hosts contain more nutrients, which could benefit offspring wasps [1], [13], [30], [35], [37], [38], and parasitoids commonly use host size as a criterion for quality [39]. Besides benefits attained on large hosts, parasitoids also incurs some risks when attacking large hosts. For *S. harmandi*, the wasp prefers to attack large larvae and gains advantages at the cost of longer dealing time, higher mortality and lower parasitism rate. All the tests herein were carried out by exposing larvae directly to the parasitoids. In the wild, however, females attack larvae concealed in wood. It cannot be excluded that the natural foraging behavior could be largely different than the one observed on ‘naked’ larvae, in which other cues may be used to detect concealed larvae. How *S. harmandi* has evolved the mechanism to measure and select large host is remained unknown and is definitely worthwhile to do further research since it is a general behavior of parasitoids.

The success of mass rearing of *S. harmandi* depends on efficient food consumption. After domestication, *M. alternatus* larvae could be an ideal host for the mass rearing of *S. harmandi*, as opposed to the currently used host, *S. populnea*, whose larvae are nearly a tenth of the size of *M. alternatus* larvae [16], [17]. Additional challenges to improve *S. harmandi*'s mass-rearing efficiency in the laboratory, and its parasitism to target-hosts in the field, need to be overcome. Future work should focus on the effects of host's immune system, and on the effects of metabolic and nutritional status on the behavioral and physiological conditioning of *S. harmandi*. Under natural conditions, selection of the targeted host developmental stage, regulation of parasitoid numbers released and introduction of food supplements, are operational factors with a potential to influence the level of biological control.

## Materials and Methods

### Experiment design and Insects

Two experiments were conducted to test the effects of host size on 1) feeding choices by female *S. harmandi*; and 2) fitness-related performance by adult and offspring females. Base stocks of *S. harmandi* for all experiments were obtained from Xishan Forest Farm (Beijing), reared on larvae of *Saperda populnea*, which is used generally as a substitute host in mass rearing of *S. harmandi*. In the laboratory, the parasitoid was reared solely on larvae of *M. alternatus* for twenty generations prior to the experiment to exclude the possible influence of the host source [17], [40]. Various sizes larvae of *M. alternatus* were collected from Zhejiang province in 2010. All larvae were stored at 8∼10°C prior to use in parasitoid rearing.

*S. harmandi* were reared individually in vials (7.5 cm in height×1.2 cm in diameter), each blocked with a tampon on the port and kept at 25±5°C, 70% RH under a LD 14:10 h. Mated female *S. harmandi* were fed on 10% honey for 5–6 days and then presented with host larvae in each vial for subsequent oviposition/feeding.

### Host size preference experiments

120 *M. alternatus* larvae were weighed and separated into large and small groups with body weight difference on the average of 229±9.8 mg (Fig. 1A). Two-choice tests were used to determine feeding preference of the two treatment groups. In a two-choice bioassay, two larvae (large and small) were put simultaneously in a glass Petri dish of 12 cm in diameter. One female wasp was put on center of each dish with a fine brush. Totally, 40 replicates were carried out. Two-choice tests were conducted in a climate chamber at 25–26°C in dark since the parasitoid forages and attacks host in concealed sites in field.

Feeding preference was expressed as the host-selecting rate of female *S. harmandi* to host at 24, 48 and 72 h. The host selection rate was defined as the proportion of females that attacked hosts with simultaneous probing, stinging and feeding behaviors up to 5 min. In a successful host selection, female *S. harmandi* walked, searched and probed throughout the arena, generally not changing positions for 24 h after making a selection.

### Host size suitability experiments

Mated female *S. harmandi* maintained on *M. alternatus* larvae were fed on 10% honey for 5–6 days and kept at 8–10°C. Females of each treatment group were placed at room temperature for half day before testing to recover activity and used only once. No-choice tests were carried out in a glass pipe (as mentioned above) and tested at 25–26°C and 14:10 h LD daylight regime.

*M. alternatus* larvae were separated into three groups by body weight, i.e., large, medium and small, respectively, as shown in Figure 1B, with about 50 replicates for each group. A larval host was offered to a mated female wasp and checked every day. Adult and its own offspring fitness consequences on each host were recorded after the female wasps oviposited on larvae and offspring completed development on the paralyzed hosts for 30–40 days. Female pre-oviposition period (days), wasp survival, parasite rate (i.e. proportion of eggs laid wasps to all alive wasps), and female fecundity (number of eggs laid per female and offspring wasp emerged) on different size of *M. alternatus* larvae were observed and recorded. The pre-oviposition periods of adult females were measured as time from wasp inoculation to first reproduction in females. Offspring performance was determined by parameters of weight of eclosing adult (mg) and survival (%) from egg to emergence and sex ratio (proportion of females). The deteriorated hosts were excluded and mortality was checked daily.

### Data analysis and statistics

Statistical analyses for this study were performed using SPSS 13.0 for Windows (SPSS Inc., Chicago, IL, USA). Chi-square test was used to compare feeding preference in dual-choice experiment, wasp mortality and parasitism rate on large and small *M. alternatus* larvae in no-choice experiments. One-way analysis of variance (ANOVA) was performed to assess the differences in adult performance (pre-oviposition period and fecundity) and offspring performance (body weight, survival and sex ratio) of *S. harmandi* and means were compared with a Bonferroni Multiple Comparison. Regression analyses were used to describe the various relationships between the body sizes of hosts parasitized, fecundity (both measured as the number of eggs laid and offspring wasps emerged), the duration of pre-oviposition, offspring parasitoid survival and body size. The data of duration of pre-oviposition and body weight were transformed by a square root transformation prior to the analysis. Percentage-based data (survival and sex ratio) were analyzed with ANOVA and means were separated with a Bonferroni Multiple Comparison after normal distribution test.
